# An overview of symbiotic and pathogenic interactions at the fungi-plant interface under environmental constraints

**DOI:** 10.3389/ffunb.2024.1363460

**Published:** 2024-10-25

**Authors:** Sunishtha Mishra, Anukriti Srivastava, Ajeet Singh, Girish Chandra Pandey, Garima Srivastava

**Affiliations:** ^1^ Department of Bioscience and Biotechnology, Banasthali Vidyapith, Rajasthan, India; ^2^ Department of Botany, Government Adarsh Girls College Sheopur, Madhya Pradesh, India

**Keywords:** fungi-plant interface, mycorrhizal relationship, restoration ecology, mutualism, antagonism

## Abstract

The complex and dynamic interactions between fungi and plants constitute a critical arena in ecological science. In this comprehensive review paper, we explore the multifaceted relationships at the fungi-plant interface, encompassing both mutualistic and antagonistic interactions, and the environmental factors influencing these associations. Mutualistic associations, notably mycorrhizal relationships, play a pivotal role in enhancing plant health and ecological balance. On the contrary, fungal diseases pose a significant threat to plant health, agriculture, and natural ecosystems, such as rusts, smuts, powdery mildews, downy mildews, and wilts, which can cause extensive damage and lead to substantial economic losses. Environmental constraints encompassing abiotic and biotic factors are elucidated to understand their role in shaping the fungi-plant interface. Temperature, moisture, and soil conditions, along with the presence of other microbes, herbivores, and competing plants, significantly influence the outcome of these interactions. The interplay between mutualism and antagonism is emphasised as a key determinant of ecosystem health and stability. The implications of these interactions extend to overall ecosystem productivity, agriculture, and conservation efforts. The potential applications of this knowledge in bioremediation, biotechnology, and biocontrol strategies emphasise the importance of adapting to climate change. However, challenges and future directions in this field include the impacts of climate change, emerging fungal pathogens, genomic insights, and the role of the fungi-plant interface in restoration ecology. Hence, this review paper provides a comprehensive overview of fungi-plant interactions, their environmental influences, and their applications in agriculture, conservation, and ecological restoration.

## Graphical Abstract

Symbiotic and pathogenic interactions at the fungi-plant interface under environmental constraints (Chang et al., 2018).

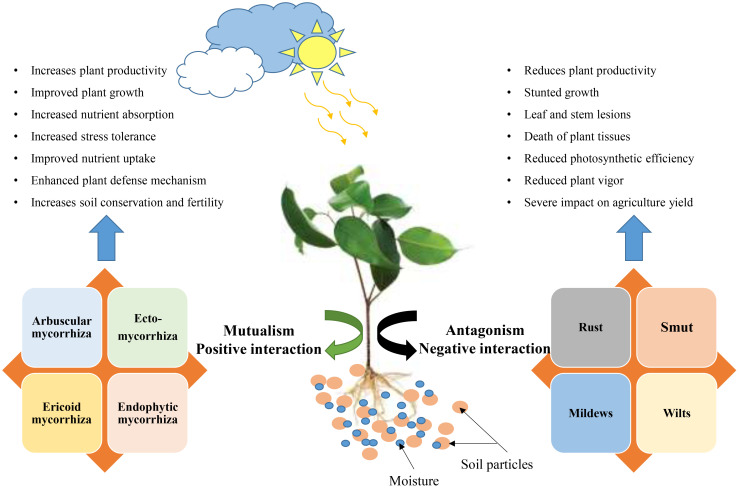

## 1 Introduction

**Figure 1:**
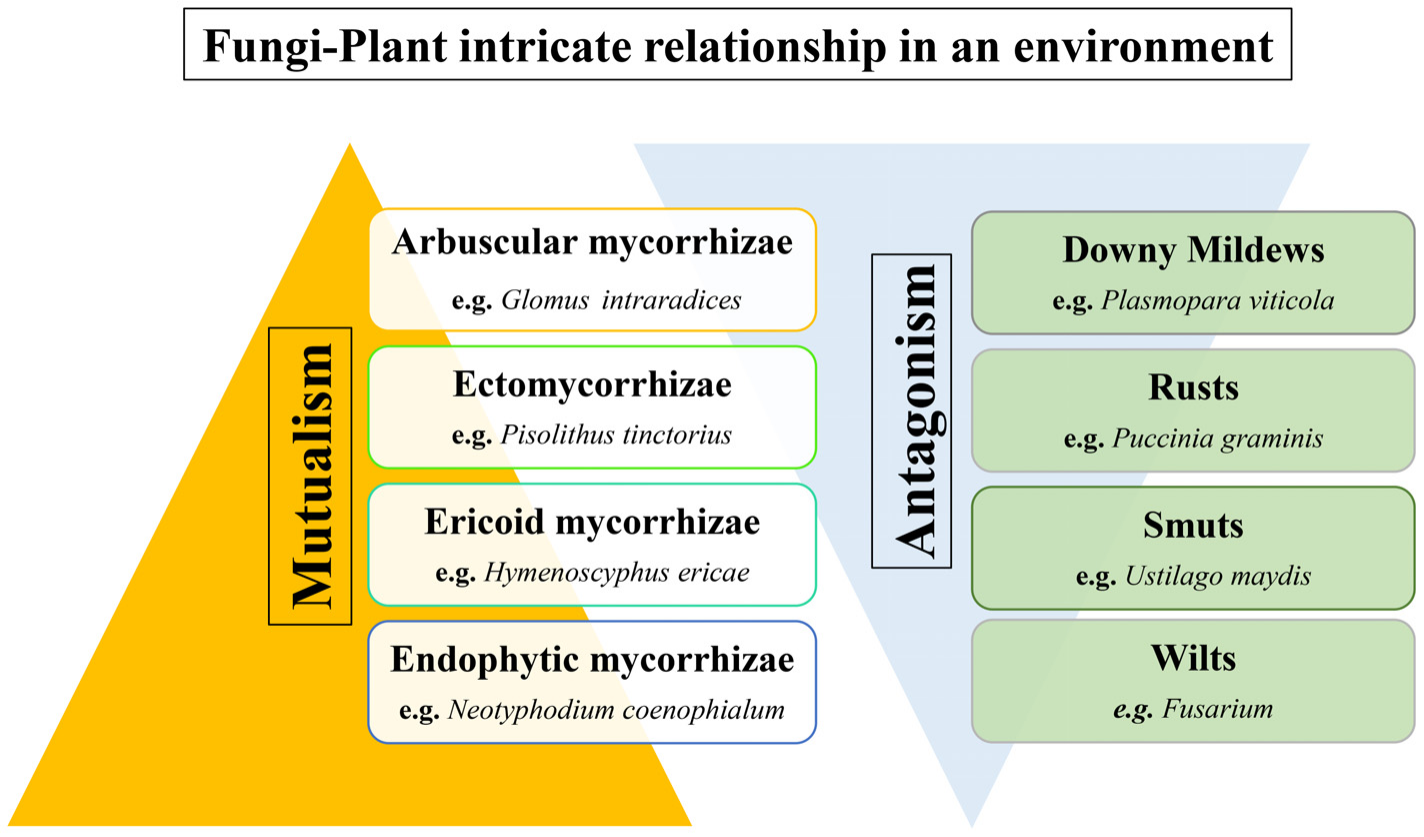
Fungi-plant intricate relationship showing both mutualistic and antagonistic behaviour on plant health.

The intricate and ever-evolving interplay between fungi and plants is a pivotal and dynamic aspect of ecological systems, where a multitude of relationships are characterised by both mutualistic and antagonistic interactions (Zeilinger et al., 2016; Balestrini, 2021; Priyashantha et al., 2023). These interactions bear profound repercussions for the vitality and adaptability of plant life, as well as for the ecological equilibrium in diverse ecosystems (Zeilinger et al., 2016; Balestrini, 2021; Figueiredo et al., 2021; Priyashantha et al., 2023), as discussed in **Figure 1**. In this review, we embark on a journey into the multitude of domains of the fungi-plant interface, meticulously delving into the intricate mechanisms and ecological implications that underlie these interactions.

Mycorrhizal symbiosis, in particular, has garnered significant attention from researchers. In this mutualistic relationship, certain fungi, predominantly arbuscular mycorrhizal fungi and ectomycorrhizal fungi, form intricate associations with plant roots. These fungi enhance the plant’s nutrient uptake, particularly phosphorus and nitrogen, by expanding the root’s absorptive surface area through the formation of mycorrhizal network structures (Lanfranco et al., 2016; Begum et al., 2019; Shi et al., 2023). In return, plants offer a steady supply of carbon compounds in the form of sugars to their fungal partners. This mutualistic exchange of resources enhances plant growth, nutrient acquisition, and stress tolerance, thereby promoting plant fitness (Stuart and Plett, 2020; Priyashantha et al., 2023).

However, not all interactions between fungi and plants are always beneficial. Antagonistic relationships in the form of pathogenic interactions often challenge the vitality of plants (Frey-Klett et al., 2011; Deveau et al., 2018; Balestrini, 2021). Many fungal species, such as rusts, smuts, and mildews, have evolved strategies to infiltrate plant tissues and exploit them as hosts. The consequences of such interactions can be detrimental, leading to reduced plant growth, low crop yield or production, and extensive ecological disruptions (Zeilinger et al., 2016; Peng et al., 2021; Mapuranga et al., 2022). Understanding the mechanisms that underpin fungi-plant pathogenesis is effective in devising strategies to mitigate the impact of such antagonistic relationships (Selin et al., 2016; Adnan et al., 2019; Dutta et al., 2023).

The modulation of these complex interactions is influenced by multiple factors. Environmental conditions such as temperature, humidity, and soil chemistry play a pivotal role in shaping the outcome of these associations (Canarini et al., 2019; Abdul Rahman et al., 2021; Rodrigues et al., 2023). Furthermore, plant genetic factors, including resistance genes, also contribute to the plant’s response to fungal partners, whether mutualistic or pathogenic. Recent research also highlighted the role of plant microinteractions in shaping the outcomes influencing fungal colonisation and plant health.

The broader implications of these interactions extend far beyond the fungus-plant interface. They have profound consequences for ecosystem dynamics. Mycorrhizal fungi not only benefit individual plants but can also influence the composition and structure of entire plant communities (Bonfante and Genre, 2010; Chen et al., 2018; Wahab et al., 2023). Furthermore, mycorrhizal networks can facilitate the transfer of nutrients between plants, connecting neighbouring individuals in complex underground exchanges.

However, the actions of pathogenic fungi can destabilise ecosystems by affecting the health and survival of key plant species, cascading through the food web, and potentially causing shifts in community composition (Gorzelak et al., 2015; Figueiredo et al., 2021).

The interactions between fungi and plants represent a captivating and intricate tapestry of mutualistic and antagonistic relationships. These associations are governed by an interplay of environmental, genetic, and microbial factors, with far-reaching consequences for plant health and ecosystem dynamics (Zeilinger et al., 2016; Alam et al., 2021; Bastías Campos et al., 2023; Priyashantha et al., 2023). With a comprehensive understanding of the mechanisms and ecological implications of these interactions, we gain valuable insights into the functioning of our natural world and pave the way for more informed strategies for the management and preservation of ecosystems. In this review, we are exploring the fungi-plant interplay both in terms of mutualism and antagonism.

## 2 Mutualistic associations

Mycorrhizal relationships are among the most intriguing and ecologically significant mutualistic associations in the complex realm of the fungi-plant interface. Mycorrhizal fungi encompass a diverse array of taxa and form mutually beneficial symbiotic partnerships with an astonishing majority of plant species, spanning the evolutionary spectrum from humble liverworts to advanced angiosperms, thus underscoring their ubiquity and central role in shaping plant life (Lanfranco et al., 2016; Chen et al., 2018; Huey et al., 2020; Wahab et al., 2023). They play a pivotal role in bolstering nutrient acquisition and overall plant fitness, as discussed in **Table 1**. These associations have evolved into several distinct types, each finely tuned to the specific requirements and ecological niches of different plant species and environments (Huey et al., 2020; Shi et al., 2023).

**Table 1:** Impact of mutualistic association of fungi-plant on the holistic growth of plant and improves stress management.

S. No.	Fungi	Host plant	Type of fungi	Impact on plant	References
1.	*Glomus intraradices*	Black gram and Maize	Arbuscular mycorrhizal fungi	Improving nutrient use efficiency also accelerates the defense response in Blackgram against *S. litura*.	Selvaraj et al., 2020
	*Rhizophagus irregularis*	Wheat			
	*Funneliformis mosseae*	Soyabean			
	*Gigaspora margarita*	Rice			
	*Claroideoglomus etunicatum*	Tomato			
2.	*Rhizophagus irregularis*	Wheat, Barley, Rice	Endophytic mycorrhizal fungi	Improved stress tolerance: Mycorrhizal associations enhance plant resilience to environmental stresses such as drought, salinity, and heavy metal toxicity.	Ghimire et al., 2020
	*Claroideoglomus* *etunicatum*	Maize			
	*Glomus mosseae*	Soyabean			
3.	*Neotyphodium coenophialum*	Tall fescue	Neotyphodium endophytes fungi	Enhanced drought resistance and improved growth: Neotyphodium endophytes enhance drought tolerance and growth in host grasses.	Freitas et al., 2020
	*Neotyphodium uncinatum*	Chewings fescue			
	*Neotyphodium huerfanoense*	Hard fescue			
	*Neotyphodium typhinum*	Strong creeping red fescue			
4.	*Neotyphodium coenophialum*	Grasses	Endophytic mycorrhizal fungi	Increased resistance to herbivores. Endophytic fungi produce compounds toxic to herbivores, enhancing plant defense mechanisms and reducing herbivory.	Lee et al., 2021; Sun and Shahrajabian, 2023
	*Neotyphodium lolii*	Tall fescue			
	*Epichloë festucae*	Ryegrass			
	*Fusarium verticillioides*	Maize			
	*Piriformospora indica*	Barley			
5.	*Phialocephala fortinii*	Wheat	Dark septate endophytes fungi	Enhanced stress tolerance and nutrient uptake: Dark Septate Endophytes (DSE) improve plant stress tolerance and nutrient uptake, particularly under harsh environmental conditions.	Malicka et al., 2022
	*Cadophora* sp.	Barley			
	*Microdochium bolleyi*	Corn			
	*Paraphoma radicina*	Rice			
	*Periconia macrospinosa*	Tomato			
6.	*Pezoloma ericae*	Calluna vulgaris	Ericoid mycorrhizal fungi	Improved nutrient uptake in acidic soils. enhancing the plant’s ability to absorb nutrients such as phosphorus and zinc.	Vohník and Réblová, 2023
	*Hymenoscyphus ericae*	Rhododendron			
	*Scytalidium vaccinii*	Blueberry			
	*Meliniomyces bicolor*	Cranberry			
	*Liriodendron maius*	Heathland plants			
7.	*Claroideoglomus* *Etunicatum*	Corn	Arbuscular mycorrhizae fungi	Enhanced nutrient uptake: Arbuscular Mycorrhizae (AM) form symbiotic relationships with plant roots, increasing the absorption surface area and facilitating nutrient exchange.	Wahab et al., 2023
	*Glomus fasciculatum*,	Barley			
	*Glomus mosseae*	Sunflower			
8.	*Pisolithus tinctorius*	Pine trees	Ectomycorrhizal fungi	Increase in vigor and stress resistance of young plants. Increased nutrient absorption.	Dyshko et al., 2024
	*Laccaria bicolor*	Oak			
	*Paxillus involutus*	Birch			
	*Thelephora terrestris*	Eucalyptus			
	*Hebeloma crustuliniforme*	Spruce			

Arbuscular mycorrhiza represents one of the most widespread and ancient forms of mycorrhizal associations. These symbiotic partnerships primarily involve fungi from the Glomeromycota phylum. Arbuscular mycorrhizal fungi (AMF), such as *Rhizophagus intraradices*, *Glomus mosseae*, *Funneliformis geosporum*, and *Claroideoglomus etunicatum*, form intricate networks within the root cells of plants, extending their hyphal structures into the surrounding soil (Berruti et al., 2016; Chen et al., 2018). This architecture dramatically increases the effective surface area for nutrient uptake of essential elements like phosphorus and nitrogen, which are frequently limiting factors in plant growth (Giovannini et al., 2020; Mondal et al., 2022; Demir et al., 2022; Wahab et al., 2023). Moreover, the formation of arbuscules within plant root cells enables the efficient exchange of nutrients between the plant and the fungus, exemplifying the intricate nature of this association (Strack et al., 2003; Begum et al., 2019).

Ectomycorrhizal relationships predominantly involve fungi from the Basidiomycota and Ascomycota groups. These mycorrhizal associations entail the formation of a sheath around the tips of plant roots, contributing to nutrient absorption and enhancing plant resistance to multiple stresses (Tibbett and Sanders, 2002; Agerer, 2006; Gil-Martínez et al., 2018). Ectomycorrhizal fungi contribute significantly to forest ecosystems as they are frequently associated with woody plant species, including many tree species. Examples of ectomycorrhizal fungi include *Pisolithus tinctorius*, *Amanita muscaria*, *Laccaria bicolor*, and *Suillus luteus*. By facilitating nutrient uptake and conferring protection against diseases and environmental pressures, ectomycorrhizal associations become integral to the health and vitality of these long-lived and ecologically influential plant species (Itoo and Reshi, 2013; Policelli et al., 2020; Liu et al., 2020).

Ericoid mycorrhizae constitute another unique variant of mycorrhizal association and are commonly found in plants like heathers and other ericaceous species. The fungi involved in ericoid mycorrhizal associations predominantly belong to the Ascomycota and Basidiomycota groups (Perotto et al., 2018; Fehrer et al., 2019; Vohník, 2020). These fungi assist plants in accessing essential nutrients, particularly in acidic, nutrient-poor soils where other nutrient acquisition strategies may prove insufficient (Cairney and Meharg, 2003; Wei et al., 2022). The fact that ericoid mycorrhizal fungi such as *Rhodotorula mucilaginosa*, *Oidiodendron maius, Meliniomyces variabilis*, and *Hymenoscyphus ericae* have the ability to break down complex organic compounds in such adverse environments showcases their importance in plant adaptation to challenging ecological niches.

Moving beyond endophytic associations represents another fascinating facet of mutualistic interactions between fungi and plants. Endophytic fungi take up residence within plant tissues, inhabiting the plant’s interior without causing apparent harm. Instead, they offer an array of benefits to their host, including increased resistance to herbivores and pathogens, improved tolerance to abiotic stress, and enhanced nutrient uptake (Alam et al., 2021; Chaudhary et al., 2022; Akram et al., 2023). Endophytic associations involve *Neotyphodium* endophytes and grasses, particularly within the Poaceae family. These endophytes produce alkaloids that deter herbivores and confer a competitive advantage to the host plant (Malinowski and Belesky, 2006; Rasmussen et al., 2008; Caradus and Johnson, 2020). The presence of *Neotyphodium* endophytes can significantly enhance the survival and vigour of grasses, which has substantial implications for both natural ecosystems and agriculture (Fadiji and Babalola, 2020).

Dark-septate endophytes (DSE) are characterised by their diverse presence in various plant species. They often enhance plant stress tolerance and nutrient uptake, making them indispensable for plant adaptation to adverse environmental conditions (Hou et al., 2020; Farias et al., 2020; Malicka et al., 2022). Porras-Alfaro and Bayman (2011) highlighted the role of DSE in helping plants thrive in high-stress environments, including those with high salinity and heavy metal contamination, such as *Phialophora fortinii, Cadophora finlandica, Darksidea* sp.*, and Periconia macrospinosa.* These fungi have demonstrated significant potential for supporting plant growth and resilience in challenging conditions, further emphasising their ecological importance.

## 3 Antagonistic interactions

Within the intricate tapestry of the fungi-plant interface, there exists a darker side characterised by antagonistic interactions between fungi and plants, as discussed in **Table 2**. This facet of the relationship involves the capacity of fungal pathogens to inflict devastating diseases upon their plant hosts. Fungal infections have profound consequences for plant health, agricultural production, and the equilibrium of natural ecosystems (Zeilinger et al., 2016; Peng et al., 2021; Priyashantha et al., 2023). Rusts and smuts are formidable adversaries belonging to the Pucciniales and Ustilaginales orders, respectively (Zuo et al., 2019; Thambugala et al., 2020; Peng et al., 2021). They inflict diseases on numerous crop plants, including staple grains such as wheat, barley, and oats. The terms “rust” and “smut” aptly describe the characteristic appearances of these diseases on plant surfaces, where they manifest as conspicuous, powdery lesions or dark, sooty pustules (Doehlemann et al., 2017; Zuo et al., 2019). These diseases are renowned for their yield losses, making them subjects of intensive study and management efforts in agriculture.

**Table 2:** Impact of antagonistic interaction between fungi-plant on the overall growth of plant species and productivity.

S.No.	Type of Fungi	Fungi Species	Host Plant	Disadvantages	References
1.	Rust Fungi	*Puccinia graminis*, *Puccinia striiformis*, *Uromyces* *appendiculatus, Melampsora* *lini, Cronartium ribicola*	Wheat Barley Oats	Severe crop yield reduction, economic losses, decreased food security.	Zuo et al., 2019
2.	Smut Fungi	*Ustilago maydis*, *Sporisorium sorghi, Ustilago avenae, Tilletia* *caries, Ustilago tritici*	Corn Sorghum Wheat Barley	Distorted plant growth, reduced yield, grain quality issues.	Ghimire et al., 2020
3.	Powdery Mildews	*Erysiphe necator*, *Podosphaera xanthii*, *Sphaerotheca pannosa*, *Blumeria graminis*, *Leveillula taurica*	Roses Cucumbers Grapes	Weakening of plants, reduced vigor, lower crop quality	Thakur et al., 2023
4.	Fusarium Species	*Fusarium* *oxysporum*, *Fusarium verticillioides, Fusarium solani*, *Fusarium* *proliferatum*	Tomatoes Bananas	Wilt, necrosis, significant yield loss, severe economic impact.	Zakaria, 2023
5	Fusarium Species	*Fusarium* *graminearum*, *Fusarium* *proliferatum*	Wheat Maize	Head blight, reduced grain quality, mycotoxin contamination, economic loss.	Ismaila et al., 2023
6.	Downy Mildew	*Plasmopara viticola*, *Peronospora farinosa, Bremia lactucae.*	Grapes Cucurbits Cucumbers, Melons Brassicas Broccoli, Cauliflower Lettuce, Spinach, Sunflowers	Leaf spots, chlorosis, wilting, defoliation, stunted growth, significant yield loss, reduced marketability of crops.	Salcedo et al., (2021)
7.	Downy Mildew	*Hyaloperonospora* *parasitica, Pseudoperonospora cubensis*	Brassicas Broccoli, Cauliflower Cucurbits Cucumbers Melons	Reduced photosynthesis, increased plant stress, premature leaf drop impaired plant development.	Salcedo et al., 2023

Powdery mildews, primarily originating from the Erysiphales order, are formidable fungal pathogens with a broad host range, infecting various plant species and targeting different parts such as leaves, stems, and flowers. The distinctive powdery white colonies on plant surfaces mark their presence (Micali et al., 2008; Kiss et al., 2020; Yeh et al., 2021). Although these fungi do not always cause catastrophic damage, they can weaken plants and reduce the quality of agricultural and horticultural products. For instance, *Erysiphe cichoracearum* affects many cucurbits, including cucumbers and melons, leading to significant yield losses (McGrath et al., 2001). *Blumeria graminis* is known for its impact on cereal crops like wheat and barley, causing severe reductions in crop yield and quality (Troch et al., 2014; Basandrai et al., 2023). *Podosphaera leucotricha* commonly infects apple and pear trees, affecting both leaves and fruits, resulting in economic losses in fruit production (Heidenreich and Turechek, 2016; Whitehead et al., 2021). Additionally, *Golovinomyces orontii* targets a wide range of ornamental plants and vegetables, including members of the Asteraceae family, such as lettuce and sunflower (Prahl et al., 2023). These examples highlight the diverse impact of powdery mildew fungi on agricultural and horticultural plants, emphasising the need for effective management strategies to mitigate their effects.

Downy mildews, belonging to the oomycetes group, represent a significant threat to agriculture due to their devastating impact on various crops, including grapes and potatoes. These pathogens are notorious for their ability to rapidly spread and infect entire harvests. They produce sporangia that can be easily dispersed by wind and water, facilitating widespread infection of new plant hosts (Thakur and Mathur, 2002; Salcedo et al., 2021). For example, *Plasmopara viticola*, the causative agent of grapevine downy mildew, can lead to severe yield losses in vineyards if not properly managed (Gessler et al., 2011; Puelles et al., 2024). *Phytophthora infestans*, responsible for potato late blight, has historically caused catastrophic famines and continues to pose a major challenge to potato production worldwide (Haverkort et al., 2009). *Peronospora destructor* targets onion crops, significantly affecting bulb quality and yield (Schwartz and Mohan, 2008; Zhou, 2023). Additionally, *Bremia lactucae* is known to infect lettuce, leading to substantial losses in both field and greenhouse settings (Lebeda et al., 2008; Macioszek et al., 2023). The extensive damage caused by downy mildew necessitates vigilant monitoring and effective management practices to mitigate their impact on agriculture.

*Fusarium* wilt, caused by various *Fusarium* species, is yet another formidable fungal disease that strikes at the heart of agriculture. This disease targets a range of economically significant crops, including tomatoes, bananas, and cotton (Dita et al., 2018; De Lamo and Takken, 2020; Duvnjak et al., 2023; Zakaria, 2023). *Fusarium* wilt is characterised by the wilting and death of affected plants (Ismaila et al., 2023). The *Fusarium* fungi often enter plants through the roots, where they disrupt water and nutrient transport systems, leading to the characteristic wilting and necrosis of plant tissues (Dita et al., 2018; Ismaila et al., 2023; Zakaria, 2023).

Antagonistic fungal interactions with plants have significant ecological and economic ramifications. They necessitate a multifaceted approach to disease management, including cultural practices, resistant plant varieties, and, in some cases, chemical control measures (Thambugala et al., 2020; He et al., 2021). Understanding the molecular and ecological intricacies of these interactions is vital for the development of more effective and sustainable disease management strategies. Furthermore, these interactions underscore the need for ongoing research to combat the ever-present threat of fungal pathogens to global agriculture and natural ecosystems.

## 4 Effect of enviornment on plant fungi interaction

**Figure 2:**
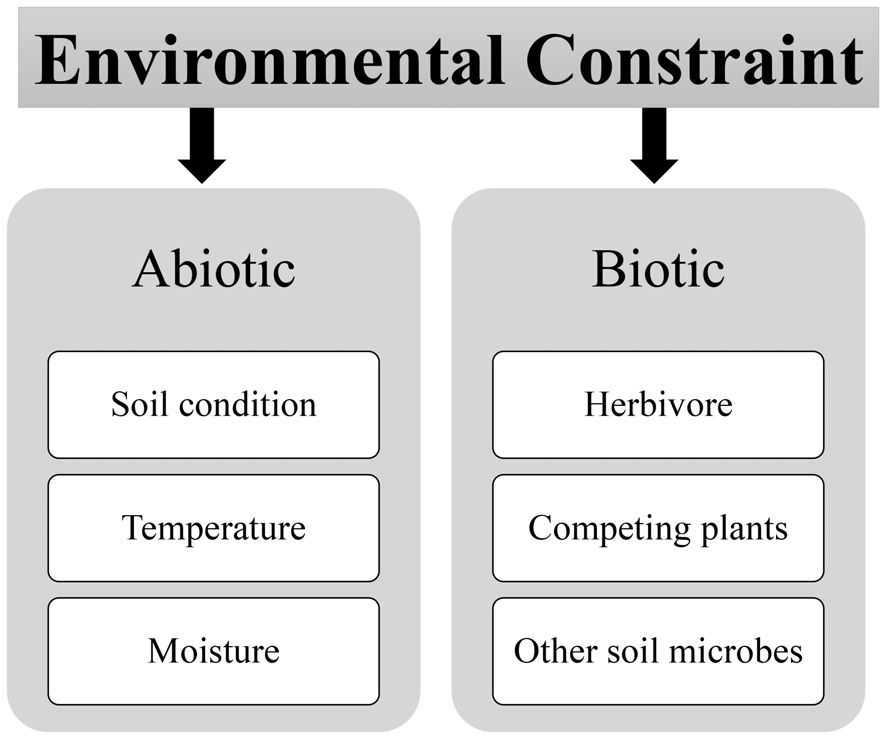
Environmental modulators of fungi-plant interface abiotic and biotic factors.

The versatile interconnection dynamic interplay between mutualistic and antagonistic interactions within the fungi-plant interface is far from static; it is profoundly influenced by a myriad of environmental factors that dictate the outcome of these associations, as shown in **Figure 2**. A comprehensive understanding of these environmental constraints is pivotal for predicting, managing, and harnessing these interactions effectively for the benefit of ecosystems and agriculture (Singh et al., 2023; Priyashantha et al., 2023). The environmental constraints are basically divided into two categories biotic and abiotic stress.

### 4.1 Abiotic factors

#### 4.1.1 Temperature

Temperature significantly influences the fungi-plant interface, affecting both mutualistic and antagonistic interactions. Temperature fluctuations impact plant susceptibility to fungal infections and the growth patterns of fungal pathogens, determining disease severity (Singh et al., 2023; Priyashantha et al., 2023). Mycorrhizal associations are also temperature-sensitive, affecting their effectiveness in various conditions. It was reported that *Glomus intraradices* perform optimally at 20–25°C, enhancing nutrient uptake and plant growth, but its efficiency decreases at higher temperatures (Tian et al., 2023). *Botrytis cinerea*, a pathogen causing grey mould, shows increased virulence at 15–20°C, making cooler climates more prone to outbreaks (Bika et al., 2021). *Piriformospora indica* promotes plant growth and stress resistance best at 25–30°C, with reduced benefits in cooler conditions necessitating temperature consideration in agricultural use (Li et al., 2023).

#### 4.1.2 Moisture

Soil moisture levels are crucial for mycorrhizal relationships and fungal pathogen activity. Arbuscular mycorrhizal fungi are less moisture-dependent but still affected, while ectomycorrhizal fungi are highly sensitive to soil water content. Moreover, moisture influences fungal spore germination, growth, and pathogenicity (Begum et al., 2019; Wahab et al., 2023). For example, the ectomycorrhizal fungus *Pisolithus tinctorius* requires high soil moisture for optimal symbiosis with pines (Dyshko et al., 2024). Rhizophagus irregularis, an arbuscular mycorrhizal fungus, thrives best in moderate moisture conditions but is adaptable to a variety of other environments (Wahab et al., 2023). The pathogen *Phytophthora infestans*, causing potato blight, thrives in moist conditions, enhancing spore germination and infection (Ivanov et al., 2021). Fusarium oxysporum, a pathogen that causes wilt, depends on sufficient soil moisture for its spores to germinate and spread (Purohit et al., 2023).

#### 4.1.3 Soil conditions

Soil characteristics, including pH, nutrient availability, and organic matter content, significantly influence the type and effectiveness of mycorrhizal associations (Huey et al., 2020). Some mycorrhizal fungi exhibit adaptations to specific soil conditions, such as ericoid mycorrhizae thriving in acidic soils (Huey et al., 2020; Wahab et al., 2023). Soil pH can dramatically affect the availability of essential nutrients like phosphorus, impacting the benefits of mycorrhizal associations. Fungal pathogens also exhibit preferences for particular soil types, affecting antagonistic interactions (Begum et al., 2019). For example, ericoid mycorrhizae such as *Rhizoscyphus ericae* thrive in acidic soils, aiding plants in nutrient-poor conditions (Yang et al., 2023). The arbuscular mycorrhizal fungus *Glomus mosseae* performs well in neutral to slightly acidic soils, enhancing phosphorus uptake (Wahab et al., 2023; Ding et al., 2024). The pathogen *Verticillium dahliae*, causing wilt, prefers alkaline soils, where it becomes more virulent (Zhang et al., 2024). The beneficial fungus *Pseudomonas fluorescens*, which can suppress soil-borne pathogens, shows enhanced activity in soils with high organic matter (Chen et al., 2023).

### 4.2 Biotic factors

#### 4.2.1 Microbes

In the soil ecosystem, various microbes interact with symbiotic and antagonistic fungi, impacting mycorrhizal associations differently. Bacteria like *Pseudomonas* spp. may compete for resources, while *Bacillus* sp. enhances plant growth. *Rhizobium* sp. influences mycorrhizal colonisation. Some fungi, like *Trichoderma* sp. and *Gliocladium* sp., inhibit mycorrhizal growth, while others, like *Penicillium* sp., either compete or cooperate. Protozoa, such as *Amoeba* sp., graze on mycorrhizal fungi. Methanotrophic archaea affect soil microbial communities. Mycoviruses infect fungi, altering their competitiveness. Understanding these interactions is crucial for comprehending the intricate web of relationships in soil ecosystems (Netherway et al., 2021; Santoyo et al., 2021). For example, *Pseudomonas fluorescens* competes with mycorrhizal fungi for nutrients, potentially reducing their effectiveness. *Bacillus subtilis* enhances plant growth and can promote mycorrhizal colonisation by producing beneficial compounds (Kulkova et al., 2023). *Rhizobium leguminosarum* positively influences mycorrhizal colonisation by improving nutrient availability through nitrogen fixation (Dhiman et al., 2024). *Trichoderma harzianum* inhibits mycorrhizal fungi through the production of antifungal compounds (Rahman et al., 2023). *Penicillium bilaii* can either compete with or assist mycorrhizal fungi in phosphate solubilisation, depending on environmental conditions (Suraby et al., 2023).

#### 4.2.2 Herbivores

Herbivores such as insects and mammals indirectly impact the fungi-plant interface by damaging plant tissues and creating entry points for fungal pathogens. This damage can render plants more susceptible to infections, particularly by fungal pathogens that exploit wounds or weaken plant defenses. Conversely, certain endophytic fungi residing within plant tissues produce compounds that deter herbivores from feeding, thus indirectly protecting their host plants (Lu et al., 2021; Chaudhary et al., 2022). These interactions highlight the interconnectedness of different biotic factors and their repercussions on the fungi-plant interface. For example, insect herbivory by caterpillars on leaves creates wounds that can be exploited by the fungal pathogen *Botrytis cinerea*, leading to increased infection rates (Ngah et al., 2024). Mammalian herbivores, such as deer, can damage bark and stems, providing entry points for fungal pathogens like *Armillaria* spp., which cause root rot (Kranjec Orlović et al., 2024). Aphid feeding can weaken plant defences, making them more susceptible to fungal infections like *Verticillium dahliae*, which cause wilt (Sun et al., 2023). Endophytic fungi such as *Neotyphodium* sp. produce alkaloids that deter herbivores like grasshoppers, thereby protecting their host plants (Lin et al., 2024). The endophytic fungus *Piriformospora indica* enhances plant resistance to herbivores by inducing systemic defence responses, reducing damage from pests like aphids (Adedayo and Babalola, 2023).

#### 4.2.3 Competing plants

The presence of other plant species can profoundly affect the fungi-plant interface. In the context of mycorrhizal fungi, competition for resources in the rhizosphere significantly influences the distribution and effectiveness of these mutualistic associations. Some plants have stronger mycorrhizal associations, leading to disparities in nutrient acquisition and competitive advantage (Figueiredo et al., 2021; Wahab et al., 2023). These dynamics impact plant community composition and ecosystem function. For example, legumes like *Trifolium repens* (white clover) often form strong mycorrhizal associations, enhancing their nutrient uptake and competitiveness (Tello-García et al., 2023). Grasses such as *Lolium perenne* (perennial ryegrass) can outcompete other plants by efficiently utilising mycorrhizal networks to access soil nutrients (Sudharsan et al., 2023). *Helianthus annuus* (sunflower) may dominate in nutrient-poor soils due to its effective mycorrhizal associations, giving it a competitive edge over less mycorrhizal-dependent species (Wahab et al., 2023). Invasive species like *Ailanthus altissima* (the tree of heaven) can disrupt local mycorrhizal networks, negatively impacting native plant species that rely on these fungi (Raspor et al., 2023). *Betula pendula* (silver birch) forms strong ectomycorrhizal associations, allowing it to compete effectively in mixed forests by accessing deep soil nutrients.

The interactions within the fungi-plant interface are intricately woven into the fabric of the natural world, and they are highly contingent upon a multitude of abiotic and biotic factors. Understanding these environmental constraints is essential for predicting the outcomes of mutualistic and antagonistic associations, enabling more effective management strategies, and shedding light on the delicate balance that governs ecological and agricultural systems (Singh et al., 2011; Balestrini, 2021). These insights are crucial for mitigating the impact of fungal diseases in agriculture and harnessing the benefits of mycorrhizal symbiosis.

## 5 The significance and challenges of fungus-plant interaction in various fields

**Figure 3:**
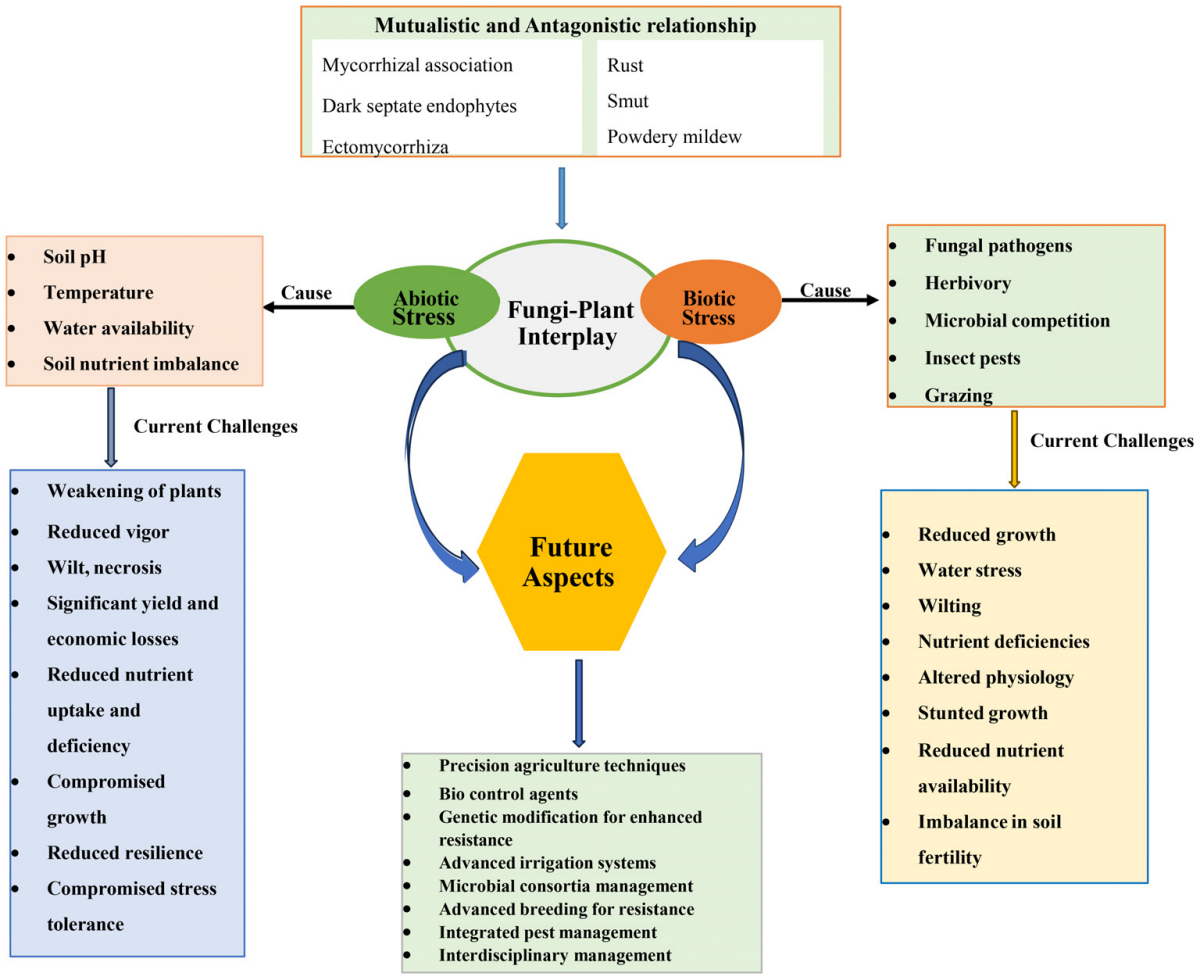
A holistic overview of fungi-plant interaction under environmental constraints, challenges and future aspects.

The fungi-plant interface represents a dynamic and ever-evolving realm where mutualistic and antagonistic interactions are perpetually at play. These interactions are emblematic of the intricate web of life that characterises the natural world. As we delve deeper into the intricacies of these associations, it becomes increasingly apparent that the equilibrium between mutualism and antagonism is a backbone in determining the health and stability of ecosystems, as shown in **Figure 3** (Zeilinger et al., 2016; Mayer et al., 2023).

### 5.1 Consequences for the ecosystem

Healthy mycorrhizal associations play a pivotal role in enhancing the overall productivity of ecosystems. They enhance nutrient cycling by improving the acquisition of vital elements, such as phosphorus and nitrogen. This nutrient cycling helps many plant communities grow and survive, not just individual plants (Begum et al., 2019; Huey et al., 2020; Priyashantha et al., 2023). These intricate networks of cooperation beneath the soil surface not only nourish individual plants but also facilitate the transfer of nutrients and signalling compounds between neighbouring plants. This interconnection influences the composition and structure of entire plant communities and has a cascading effect on the ecological balance of ecosystems (Yu et al., 2022; Shi et al., 2023).

On the contrary, outbreaks of fungal diseases can have devastating consequences for plant populations and the intricate food chains that depend on them. Such diseases can lead to a decline in plant species, reducing food resources for herbivores and, in turn, impacting their predators. These disruptions can reverberate through the entire ecosystem, leading to imbalances in predator-prey relationships and ultimately affecting ecosystem stability (Godfray et al., 2016; Almeida et al., 2019; Priyashantha et al., 2023). The consequences of these interactions emphasise the intricate interdependency within natural systems and the critical role of the fungi-plant interface in shaping the health and resilience of ecosystems.

### 5.2 Agriculture

In the agricultural domain, comprehending and managing the fungus-plant interface holds significant value. Farmers and agronomists can leverage mycorrhizal associations to enhance crop yields and reduce dependence on chemical fertilisers. These symbiotic interactions substantially augment nutrient uptake in crops, fostering improved plant growth and development. By facilitating the acquisition of essential nutrients, such mutualistic associations contribute to sustainable farming practices (Berruti et al., 2016; Wahab et al., 2023).

At the same time, developing strategies to mitigate fungal pathogens’ impact on crops is pivotal for preserving crop yields. Fungal diseases can cause considerable yield losses and economic setbacks in agriculture. Management techniques typically involve fungicide use, breeding for disease resistance, and cultural practices. Achieving a balance between harnessing the benefits of mutualistic associations and safeguarding crops from antagonistic interactions remains an ongoing challenge in the agricultural sector (Wahab et al., 2023).

Understanding the fungi-plant interface has sparked innovative applications in biotechnology. Mycorrhizal inoculants, including species such as *Rhizophagus irregularis, Funneliformis mosseae*, and *Gigaspora margarita*, form symbiotic partnerships with soybean roots, enhancing nutrient uptake, particularly phosphorus (Wahab et al., 2023). Research confirms that these inoculants significantly increase soybean growth, nitrogen fixation, and nutrient uptake, leading to improved yield and quality (Leite et al., 2024). Synergistic effects with nitrogen-fixing bacteria such as Bradyrhizobium japonicum further enhance plant biomass and nitrogen content (Leite et al., 2024). Mixed inoculant formulations, incorporating multiple mycorrhizal fungi species, have demonstrated enhanced growth and stress tolerance in soybeans compared to single-species inoculants (Igiehon and Babalola, 2021). Additionally, mycorrhizal inoculants have shown promising results in various other crops. In maize cultivation, *Rhizophagus irregularis* has been found to improve nutrient uptake and increase plant growth. Similarly, in wheat, mycorrhizal inoculants enhance phosphorus acquisition and improve crop yield (Wu et al., 2022; Wahab et al., 2023). Moreover, in leguminous crops like common bean, mycorrhizal symbiosis enhances nitrogen fixation and promotes plant growth (Yu et al., 2024). These findings underscore the broad applicability and effectiveness of mycorrhizal inoculants across different crops, reinforcing their role in sustainable agriculture practices aimed at reducing chemical fertiliser dependence and improving overall crop productivity.

Mycorrhizal inoculants have undergone testing across various crop systems, yielding promising results. These formulations contain carefully selected mycorrhizal fungal species that establish beneficial associations with crops, enhancing access to vital nutrients like phosphorus and nitrogen. By bolstering nutrient uptake, these inoculants can amplify crop yields and elevate agricultural product quality (Berruti et al., 2016; Emmanuel and Babalola, 2020; Etesami et al., 2021). This approach resonates with precision agriculture principles, emphasising efficient input utilisation to optimise productivity while minimising environmental impact (Wahab et al., 2023).

### 5.3 Conservation

In the realm of conservation biology, maintaining the equilibrium between mutualistic and antagonistic interactions in natural ecosystems is crucial for the preservation of biodiversity. Many plant species rely on mycorrhizal relationships for their growth and survival. Disruptions in these associations can have far-reaching consequences, particularly for rare and endangered species. The ghost orchid (*Dendrophylax lindenii)*, which is native to the nutrient-poor swamps of Florida and Cuba, depends heavily on its mycorrhizal associations with specific fungal partners to thrive. Mycorrhizal symbiosis can be especially vital for plants in nutrient-poor environments, where they depend on their fungal partners to survive (Begum et al., 2019; Li et al., 2021). Conserving the diversity of mycorrhizal fungi and understanding their relationships with plants is pivotal for the protection of plant species and the maintenance of ecological balance in natural ecosystems.

The equilibrium between symbiotic relationships is crucial for ecosystem health and stability, impacting organisms and trophic networks alike (Bahram and Netherway, 2022; Shi et al., 2023). In agriculture, it supports sustainable practices and crop resilience, while in conservation biology, it emphasises the need to protect trophic interdependencies. Studying and utilising the mycorrhizal-plant interface holds promise for ecological restoration and agricultural sustainability in a changing world.

A recent study by Bahram and Netherway (2022) exemplifies the impact of mycorrhizal associations on plant nutrient acquisition and stress tolerance. They found that specific mycorrhizal fungi, such as *Rhizophagus irregularis*, enhance phosphorus uptake in plants, thereby increasing nutrient acquisition efficiency and improving resilience to environmental stresses like drought, as well as their role in shaping ecosystem dynamics and resilience (e.g., Shi et al., 2023).

### 5.4 Bioremediation

Mycorrhizal fungi, particularly arbuscular mycorrhizae, are integral to soil health and have garnered attention for their potential in bioremediation efforts. One of the most notable applications is in the restoration of degraded ecosystems and the remediation of contaminated soils (Asmelash et al., 2016; Sharma et al., 2021; Wahab et al., 2023). For instance, *Rhizophagus irregularis* has been extensively studied for its role in enhancing the uptake of pollutants, such as heavy metals, by plants, thereby aiding in the detoxification and removal of these contaminants from the soil (Boorboori and Zhang, 2022; Ma et al., 2022; Fall et al., 2022). Additionally, *Glomus mosseae*, *Funneliformis mosseae*, and *Gigaspora margarita* have also shown promising potential in bioremediation efforts through their ability to improve plant tolerance to environmental stresses and facilitate the degradation of various soil pollutants (Smith et al., 2018; Zhang et al., 2020; Li et al., 2023).

Arbuscular mycorrhizal fungi have been identified as forming symbiotic associations with plants thriving in polluted environments, enhancing the plant’s capability to uptake and immobilise heavy metals within their tissues (Begum et al., 2019; Boorboori and Zhang, 2022). These fungi establish a mycorrhizal network that extends the root system and acts as an efficient conduit for metal translocation from the soil to the plant’s above-ground parts. This mechanism can substantially decrease metal concentrations in the soil, thereby reducing its toxicity and aiding in ecosystem restoration (Smith et al., 2011; Bücking and Kafle, 2015). Additionally, the accumulation of heavy metals within plant biomass is of interest for phytoremediation, a strategy utilising hyperaccumulating plants to extract metals from polluted soils for subsequent harvesting and processing (Yan et al., 2020; Nedjimi, 2021; Kafle et al., 2022). *Glomus intraradices* have shown promise in facilitating the sequestration of heavy metals within plant tissues, thereby aiding in the remediation of contaminated soils (Boorboori and Zhang, 2022).

### 5.5 Biocontrol

Beneficial fungi, like *Trichoderma* species, are widely employed in biocontrol strategies against plant pathogens. Serving as natural antagonists, these biocontrol agents actively suppress the growth of harmful fungi, safeguarding plants from diseases and reducing reliance on chemical pesticides (Sun et al., 2023). *Trichoderma*, renowned for its mycoparasitic activity, parasitises various plant pathogens by producing enzymes and secondary metabolites that inhibit their growth and infection processes (Hermosa et al., 2012). By harnessing these natural antagonists, farmers can minimise agriculture’s environmental footprint, decrease pesticide residues in food, and promote the health and sustainability of agricultural ecosystems.

Understanding the fungi-plant interaction has led to practical applications with broad implications. From bioremediation to biocontrol, these applications demonstrate how insights into fungal-plant interactions can address crucial challenges in environmental restoration, agriculture, and sustainable land management. As we continue to explore and unlock the potential of these interactions, we pave the way for more efficient, eco-friendly, and sustainable solutions to pressing planetary issues.

### 5.6 Climate change impact

The fungi-plant interface faces significant challenges amidst the accelerating pace of global climate change. Escalating temperatures, shifting precipitation patterns, and heightened atmospheric carbon dioxide levels have the potential to disrupt established mutualistic and antagonistic interactions (Bennett and Classen, 2020; Duarte and Maherali, 2022). Such disruptions can affect fungal distribution, the virulence of pathogens, and the adaptability of mycorrhizal fungi to changing conditions. For instance, beneficial fungi like *Trichoderma harzianum*, known for its biocontrol properties, may encounter difficulties in suppressing plant pathogens due to these climatic shifts. Similarly, *Rhizophagus irregularis* and *Glomus intraradices*, both arbuscular mycorrhizal fungi that significantly enhance nutrient uptake in plants, are sensitive to temperature and moisture changes, potentially affecting their efficiency and the health of the plants they associate with (Ghorbanpour et al., 2018; Tyśkiewicz et al., 2022). On the pathogenic side, fungi such as *Fusarium oxysporum* could become more virulent under changing climate conditions, posing greater threats to crop health and agricultural productivity (Chakrapani et al., 2023; Ekwomadu and Mwanza, 2023). Addressing these challenges requires a deeper understanding of how environmental changes influence these intricate interactions and the development of strategies to enhance ecosystem resilience, ensuring the sustainability of both agricultural and natural ecosystems.

### 5.7 Emerging pathogens

The emergence of new fungal pathogens poses a continuous threat to agricultural and natural ecosystems, leading to disease outbreaks and economic losses. Effective mitigation requires identifying these pathogens, understanding their biology and host interactions, and integrating pathogen surveillance into disease management strategies for early detection and response. Notable examples of harmful fungi include *Magnaporthe oryzae*, which causes rice blast disease; *Phytophthora infestans*, responsible for late blight in potatoes and tomatoes; *Puccinia graminis* sp. *tritici*, which causes wheat stem rust; and Batrachochytrium dendrobatidis, which affects amphibians. These examples highlight the broader ecological threats posed by fungal diseases (Zhou et al., 2019; Ristaino et al., 2021). Addressing these emerging threats is crucial for protecting both agricultural productivity and ecosystem health.

## 6 Future directions

### 6.1 Harnessing fungal diversity

Exploring the vast diversity of fungi holds promise for uncovering novel beneficial species that could be utilised in agriculture and environmental restoration. Research efforts should focus on characterising fungal communities and their potential roles in enhancing plant health, soil fertility, and ecosystem resilience. For instance, *Beauveria bassiana* is valued for its entomopathogenic properties, making it a powerful biocontrol agent against insect pests. *Penicillium bilaii* has demonstrated the ability to solubilise phosphate, improving soil fertility and plant nutrient availability (Frąc et al., 2018; Noorjahan et al., 2022). *Rhizophagus irregularis* and *Glomus intraradices* are notable mycorrhizal fungi that enhance nutrient uptake and support robust plant growth (Kakabouki et al., 2021; Roussis et al., 2022). Additionally, *Trichoderma harzianum* is known for its ability to suppress plant pathogens and promote overall plant health (Zin and Badaluddin, 2020; Yao et al., 2023). By studying these and other fungi, we can develop innovative strategies to boost agricultural productivity and support effective ecosystem restoration, ensuring sustainable and resilient environments.

### 6.2 Restoration ecology

Restoration ecology increasingly relies on mycorrhizal fungi to restore soil health and enhance the success of ecological restoration projects. These fungi facilitate the establishment and growth of native plant species in degraded and nutrient-poor soils. For example, *Glomus intraradices* and *Rhizophagus irregularis* are commonly used to improve plant nutrient uptake and soil structure (Boorboori and Zhang, 2022). Future directions in restoration ecology involve the targeted use of mycorrhizal inoculants, such as *Pisolithus tinctorius*, to accelerate ecosystem recovery and enhance resilience to environmental stressors (Usman et al., 2021; Atala et al., 2023). Additionally, fungi like *Scleroderma citrinum* are being studied for their role in establishing mycorrhizal networks that support plant communities in challenging environments (Rajapitamahuni et al., 2023). Understanding plant-specific mycorrhizal associations informs tailored restoration approaches for diverse ecosystems and habitats, ensuring more effective and sustainable restoration outcomes.

### 6.3 Precision agriculture integration

Integrating fungal knowledge into precision agriculture practices can optimise resource utilisation and minimise environmental impacts. By leveraging insights into fungi-plant interactions, farmers can tailor management practices to specific crop and soil conditions, enhancing productivity while reducing inputs such as fertilisers and pesticides. For example, *Rhizophagus irregularis* can improve nutrient uptake in crops, reducing the need for chemical fertilisers (Roy and George, 2020; Iqbal et al., 2023; Yadav et al., 2023; Ahmed et al., 2024). *Trichoderma harzianum* is effective in controlling soil-borne diseases, decreasing reliance on chemical pesticides (Yao et al., 2023). *Penicillium bilaii* helps solubilise phosphate in the soil, making this essential nutrient more available to plants and reducing the need for phosphate fertilisers (Sánchez-Esteva et al., 2016). Additionally, *Beauveria bassiana* can be used as a biocontrol agent against insect pests, providing an environmentally friendly alternative to chemical insecticides (Bamisile et al., 2021; Furuie et al., 2022; Islam et al., 2023). By integrating these fungi into precision agriculture, farmers can achieve higher crop yields and healthier soils with lower environmental footprints.

### 6.4 Biotechnological innovations and genomic insights

Advancements in genomics provide deep insights into the genetic and molecular mechanisms underlying beneficial and pathogenic interactions within the fungi-plant interface. Understanding these genetic bases allows for the development of targeted strategies to manage both mutualistic and antagonistic associations (Sharma et al., 2020; Diwan et al., 2022; Müller et al., 2023). For instance, identifying genes associated with mycorrhizal symbiosis can inform crop breeding for enhanced compatibility with fungi like *Rhizophagus irregularis*, thereby improving nutrient uptake. Similarly, genomic insights into the virulence factors of pathogens such as *Magnaporthe oryzae*, which causes rice blast disease, can facilitate the development of disease-resistant rice varieties. Research on *Fusarium graminearum* responsible for *Fusarium* head blight in cereals has led to the identification of genes involved in toxin production, aiding in the breeding of resistant crops (Mateus et al., 2020; Guigard et al., 2023; Priyashantha et al., 2023). Additionally, understanding the genetic pathways of beneficial fungi like *Trichoderma harzianum* has paved the way for novel biocontrol agents that can effectively suppress plant pathogens (Collinge et al., 2022; Yao et al., 2023). These genomic advancements are crucial for developing innovative solutions in agriculture and ecosystem management.

Continued advancements in biotechnology offer opportunities to develop novel tools and products for managing fungal interactions in agriculture and environmental conservation. Engineered mycorrhizal fungi such as *Glomus intraradices* with enhanced nutrient uptake capabilities can significantly boost crop yields while reducing fertiliser dependence (Odoh et al., 2020; Afridi et al., 2022; Sun et al., 2023). *Trichoderma viride*, a natural biocontrol agent, can be further optimised to more effectively target specific plant pathogens, minimising the need for chemical pesticides (Lahlali et al., 2022; Yao et al., 2023). *Metarhizium anisopliae*, known for its entomopathogenic properties, can be engineered to improve its efficacy against a broader range of insect pests, providing a sustainable alternative to chemical insecticides (St. Leger and Wang, 2020; Bamisile et al., 2021). Additionally, *Aspergillus niger* can be modified to enhance its ability to produce organic acids that aid in soil nutrient solubilisation, ensuring better nutrient availability in soils (do Nascimento et al., 2021). These biotechnological innovations have the potential to revolutionise sustainable farming and ecosystem restoration practices, leading to more resilient agricultural systems and healthier environments.

### 6.5 Climate resilience strategies

Developing climate-resilient agricultural and ecological systems requires integrating fungus- and plant-based solutions. Research efforts should focus on identifying fungal species and traits that confer resilience to climate change stressors, such as drought and heat. For example, *Rhizophagus irregularis* is known for its ability to enhance plant drought tolerance by improving water uptake (Poudel et al., 2021; Verma et al., 2022). *Pisolithus tinctorius* is another mycorrhizal fungus that can help plants withstand extreme heat and poor soil conditions. *Trichoderma harzianum* not only acts as a biocontrol agent but also promotes plant growth under stressful environmental conditions (Baptista et al., 2021; Tyśkiewicz et al., 2022). Additionally, *Piriformospora indica* has been shown to increase plant resistance to both drought and salinity (Boorboori and Zhang, 2022; Li et al., 2023). Harnessing these fungi in breeding programmes and ecosystem restoration initiatives can enhance the adaptability and sustainability of agricultural and natural systems, ensuring their resilience in the face of climate change.

### 6.6 Interdisciplinary collaboration

Addressing the challenges and opportunities of the fungi-plant interface requires interdisciplinary collaboration among scientists, policymakers, farmers, and conservationists. By working together, we can develop comprehensive strategies to utilise fungal diversity for sustainable agriculture and ecosystem management, ensuring food security and environmental sustainability. For example, *Piriformospora indica* enhances plant growth and stress tolerance under drought and salinity conditions. *Serendipita vermifera* improves nutrient uptake and promotes root growth, benefiting agricultural productivity (Jeger et al., 2021; Bouri et al., 2023). *Hirsutella thompsonii* serves as a biocontrol agent against mite pests, reducing the need for chemical treatments (Kumar, 2010; Palevsky et al., 2022). *Mortierella elongata* contributes to soil health by decomposing organic matter and enhancing soil nutrient cycling (Li et al., 2018; Zhang et al., 2020). These examples demonstrate the potential of fungal-based solutions to address global environmental challenges effectively through collaborative efforts.

## 7 Conclusion

This review has highlighted the intricate and dynamic nature of interactions at the fungus-plant interface, underscoring their critical role in ecological and agricultural systems. Mutualistic relationships, particularly mycorrhizal associations, are pivotal for enhancing plant nutrient acquisition, stress tolerance, and overall ecosystem productivity. These beneficial interactions are essential for maintaining ecological balance and supporting sustainable agricultural practices. However, pathogenic fungi pose significant threats to plant health, leading to substantial agricultural losses and ecosystem disruption. The competition between these antagonistic and mutualistic interactions is profoundly influenced by environmental constraints such as temperature, moisture level, soil composition, competing plants, and other microorganisms. Rising temperatures, altered precipitation patterns, and increased atmospheric CO_2_ levels can disrupt established fungi-plant relationships, potentially leading to increased pathogen virulence and decreased efficiency of beneficial fungi. Future research should focus on integrating genomic insights to unravel the genetic and molecular mechanisms governing these interactions. Such knowledge can inform the development of crops with enhanced resistance to pathogens and improved compatibility with beneficial fungi. Additionally, applying this understanding to restoration ecology can enhance the success of efforts aimed at rehabilitating degraded ecosystems by leveraging the symbiotic potential of mycorrhizal fungi. Through interdisciplinary collaboration and innovative research, we can develop strategies that support the health and productivity of both natural and managed ecosystems, ultimately contributing to global food security and environmental conservation.
